# A Case Report of Thyroid Storm with Cardiovascular Collapse After Propranolol Administration

**DOI:** 10.5811/cpcem.41826

**Published:** 2025-08-18

**Authors:** Mark Ringer, Tammy Phan, Emmelyn J. Samones, Brian Wolk

**Affiliations:** * Loma Linda University Medical Center, Department of Emergency Medicine, Loma Linda, California; † Pomona Valley Hospital Medical Center, Department of Emergency Medicine, Pomona, California

**Keywords:** case report, propranolol, thyroid storm, appendicitis, beta blockers

## Abstract

**Introduction:**

Thyroid storm is a rare, life-threatening emergency with a 3.6–17% mortality rate despite proper management. Elevated levels of circulating thyroid hormones can increase metabolic demand, leading to adverse effects on multiple organ systems, particularly critical cardiovascular complications such as cardiomyopathy, myocardial infarction, ventricular arrhythmias, or coronary vasospasm. Precipitating factors can include infection, surgery, or trauma, with infection being the most common. The potential for cardiovascular mortality is high. While beta blockers are key for treatment, they can potentially reduce necessary cardiac output, risking hemodynamic collapse. Traditionally, propranolol has been recommended. We report a rare case of an adolescent experiencing cardiac arrest after propranolol administration in thyroid storm and acute appendicitis.

**Case Report:**

A 17-year-old female with a history of Grave disease, non-adherent to methimazole, underwent evaluation and treatment of thyroid storm and concomitant acute appendicitis. Aggressive initial treatment measures were started, including intravenous and oral propranolol. She went into cardiac arrest approximately six hours after initial medication administration, with subsequent return of spontaneous circulation achieved. The patient underwent aggressive resuscitation and multidisciplinary management. She had a prolonged course in the intensive care unit and was ultimately discharged from the hospital approximately three weeks later.

**Conclusion:**

Beta-blocker use in the management of thyrotoxicosis can potentially cause cardiovascular collapse. We suggest consideration of shorter acting beta blockers, such as esmolol or landiolol.

## INTRODUCTION

Thyroid storm is a rare but serious emergency. Even when managed appropriately, the mortality rate is 3.6–17%.^1^ Thyroid storm usually occurs in patients with underlying hyperthyroidism, such as Grave disease.^1^ Symptoms typically involve hyperthermia, tachycardia, central nervous system dysfunction, heart failure, and gastrointestinal symptoms such as nausea, vomiting, and diarrhea. Symptoms arise from a hypermetabolic state resulting from elevated levels of circulating thyroid hormone.^2^,^3^ Typical precipitating factors include infection, cardiac ischemia, surgery, or trauma, with infection the most common.^4^ Concomitant pathology may also create challenges for identification and treatment of thyroid storm, such as occurred in our case.

Cardiovascular mortality is high in the acute phase of thyroid storm.^5^ Controlling tachycardia early in the disease course may prevent permanent cardiac damage.^4^ Beta blockers are a mainstay of treatment for thyrotoxicosis and thyroid storm; however, in patients with heart failure, beta blockade can profoundly decrease cardiac output. Other reports document cardiovascular collapse after receipt of beta blockers.^5^–^8^ To date, the youngest patient reported in the literature with cardiovascular collapse after beta blocker administration for thyroid storm management was 32 years of age.^6^ We present a rare case of an adolescent patient who experienced cardiac arrest after propranolol administration in the setting of thyroid storm and concomitant acute appendicitis.

## CASE REPORT

A 17-year-old non-pregnant female, with a history of Grave disease non-adherent on methimazole, presented to an outside hospital with concern for abdominal pain. The patient weighed 53 kilograms, with initial vital signs showing a blood pressure of 92/64 millimeters of mercury; heart rate (HR) 145 beats per minute (bpm); respiratory rate, 35 breaths per minute; and oxygen saturation of 97%, with a temperature of 37.1 °Celsius. The patient was noted to be in mild distress on initial examination but alert and oriented to person, place, and time. She also had right lower quadrant tenderness to palpation and was found to have acute appendicitis on computed tomography (CT).

The patient was tachycardic and mildly hypotensive, with thyroid-stimulating hormone less than 0.015 milli-international units per milliliter (mIU/mL) (reference range: 0.465–4.680 mIU/mL), and free thyroxine (T4) greater than 6.99 nanograms per deciliter (ng/dL) (0.78–2.19 ng/dL), raising the concern for thyrotoxicosis. The patient was initially given 1.5 liters (L) of isotonic intravenous (IV) fluid, propranolol 0.5 milligrams (mg) IV push (IVP), hydrocortisone 300 mg IVP, and methimazole 20 mg by mouth. Later, the patient was given additional doses of propranolol: 1 mg IVP and 60 mg orally (PO). She also received potassium chloride 40 milliequivalents PO and calcium gluconate 1 gram (g) IV piggyback.

According to transfer records, the outside facility did not have potassium iodide available to administer. Due to concern for suspected thyroid storm and acute appendicitis, arrangements were made to transfer the patient to our tertiary-care center. The patient’s initial blood pressure improved and stabilized after IV fluid administration. However, prior to transfer, she developed worsening hypotension and lethargy, which did not improve with an additional 2 L of IV crystalloid. A norepinephrine infusion was started at 18 micrograms per hour (mcg/hr) and continued during transport, with an increased rate of 22 mcg/hr on arrival. The patient’s blood glucose was 137 mg/dL (74–106 mg/dL) at the transferring facility, just prior to transport.

On arrival at the tertiary center, the patient was initially awake and alert but was noted to be suddenly unresponsive 10 minutes later. She was found to be pulseless at that time, and cardiopulmonary resuscitation (CPR) was initiated. The norepinephrine drip was paused, and the patient was given a total of four doses of epinephrine 1 mg IVP during the code, along with one dose of atropine 1 mg IVP. The patient’s blood glucose was 27 mg/dL, and she received an initial dose of dextrose 25 g IVP with subsequent improvement of the blood glucose. She was intubated during the code and was noted to be in asystole on the first three rhythm checks. Return of spontaneous circulation was achieved after four rounds of CPR. The norepinephrine infusion was restarted, and an epinephrine infusion was added at 5 mcg/minute.

*CPC-EM Capsule*

What do we already know about this clinical entity?

*Thyroid storm is a life-threatening condition that requires early detection and prompt management. Beta blockers are a key component to the treatment regimen*.

What makes this presentation of disease reportable?

*We present a case of a pediatric patient that suffered cardiac arrest in the setting of thyroid storm, possibly secondary to propranolol toxicity*.

What is the major learning point?

*Judicious use of beta blockers in the management of thyroid storm is paramount, with consideration toward use of short-acting alternatives to propranolol*.

How might this improve emergency medicine practice?

*Careful and appropriate use of beta blockers in treatment of thyroid storm can potentially reduce complications and improve overall morbidity and mortality*.

**Image 1:**
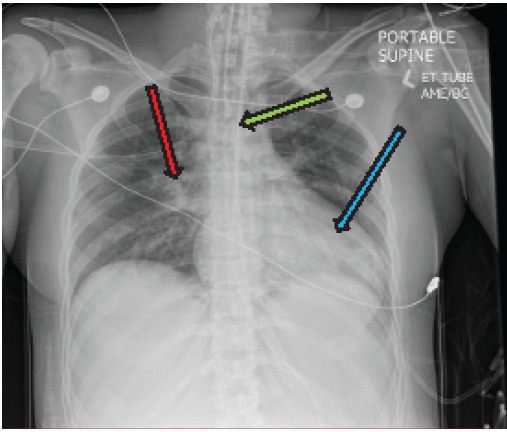
Anteroposterior chest radiograph taken in the supine position showing cardiomegaly (blue arrow) and perihilar airspace disease (red arrow), raising concern for pulmonary edema. The endotracheal tube is also seen post-intubation terminating 4.3 centimeters above the carina (green arrow).

Central venous access and arterial line was placed for blood pressure monitoring. The patient developed profound tachycardia to 170 bpm with intermittent runs of ventricular tachycardia. Magnesium 2 g IV was given with improved HR and resolution of the ventricular tachycardia. Recurrent hypoglycemia was also noted, requiring multiple additional boluses of dextrose. Glucagon was administered to circumvent the possible effects of propranolol, given the patient’s cardiovascular collapse and recurrent hypoglycemia. Due to a hospital glucagon shortage, the patient received 1 mg IV bolus followed by 2 mg/hour for five hours only. Endocrinology was consulted for management, and the patient was given hydrocortisone 100 mg IVP. Potassium iodide and methimazole were also recommended; however, these were not given in the emergency department due to a delay in availability from the hospital pharmacy. Chest radiograph was notable for cardiomegaly, with possible mild pulmonary congestion (Image 1).

The patient was admitted to the pediatric intensive care unit and started on broad-spectrum IV antibiotics: ceftriaxone, metronidazole, and vancomycin. Toxicology was consulted due to concern for beta-blocker toxicity, and the patient was also given multiple doses of calcium. Milrinone was initiated for cardiovascular support, and high-dose insulin was considered but not given for treatment of beta-blocker toxicity. Echocardiogram noted an ejection fraction of 35%, with peak troponin T, high-sensitivity 42 ng/L (≤ 13 ng/L) and pro-B-type natriuretic peptide > 70,000 picograms (pg) per mL (30–125 pg/mL). The patient progressively improved with aggressive diuresis and continued cardiovascular support.

**Image 2:**
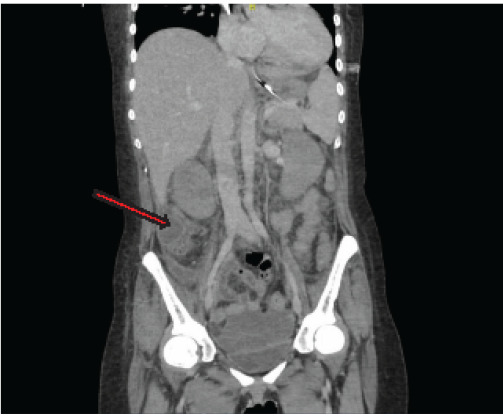
Computed tomography of the abdomen and pelvis with intravenous contrast showing perforated appendicitis with multiple peripherally enhancing fluid collections seen within the right paracolic gutter as well as the pelvis, the largest measuring up to 8.4 centimeters (red arrow). There is associated regional fat stranding and a small volume of free fluid.

Repeat echocardiogram three days later showed normal systolic function. She was extubated on hospital day 5 and was able to tolerate a regular diet. Inotropes and pressors were also weaned and discontinued on day five. Given the patient’s overall instability upon admission, pediatric surgery recommended medical management of the patient’s appendicitis. A repeat CT of the abdomen and pelvis with IV contrast on hospital day 7 showed evidence of perforated appendicitis with multiple fluid collections concerning for abscess formation (Image 2).

Three intraperitoneal drains were placed by interventional radiology on hospital day 8, and the patient was continued on IV antibiotics after abscess fluid cultures grew *Pseudomonas aeruginosa*. She also required continuous renal replacement therapy for acute kidney injury, which was transitioned to hemodialysis. Per endocrinology recommendations, the patient was continued on stress-dose hydrocortisone, which was stopped on hospital day 9. Methimazole was held initially due to elevated liver enzymes and concern for acute liver injury but was started on hospital day 7 at the patient’s home dose of 5 mg PO daily. Potassium iodide was considered, but ultimately never given due to acute kidney injury and eventual improvement in thyroid labs. Cholestyramine was initiated on hospital day 3 and discontinued on hospital day 10. Three weeks after the patient was admitted, she was discharged home on oral antibiotics. She required continued hemodialysis in the outpatient setting for approximately six weeks.

## DISCUSSION

Thyroid storm can be difficult to identify given the broad range of effects. End-organ damage in the setting of thyrotoxicosis is the key feature. The Burch-Wartofsky point scale can be used to gauge the severity of illness with a suspected thyrotoxic state. A score of < 25 indicates that thyroid storm is unlikely, 25–44 suggests impending thyroid storm, and > 45 indicates severe illness and suspected thyroid storm.^9^ According to transport records, the patient had an initial score of 70 prior to transfer.

The tenets of management are as follows: 1) giving supportive care and management of precipitating factors; 2) inhibiting the synthesis and release of thyroid hormone; and 3) controlling peripheral effects of the circulating hormones.^4^ First-line therapies typically involve thionamides such as propylthiouracil (PTU) or methimazole (MMI). Recent data suggest similar effectiveness and no difference in adverse events and in-hospital mortality between PTU and MMI.^1^ Iodine supplementation given after thionamide administration also inhibits thyroid hormone synthesis and is given as potassium iodide in either Lugol solution or saturated solution potassium iodide. Both glucocorticoids and beta blockers reduce the peripheral conversion of T4 to triiodothyronine (T3). Cholestyramine may improve gastrointestinal clearance of thyroid hormone. In severe refractory cases, plasmapheresis may be considered to remove circulating thyroid hormones from the plasma.^1^,^3^

Cardiopulmonary manifestations of thyroid storm are the most common cause of mortality. Ventricular arrhythmias are typically the proximate cause of cardiac arrest.^2^ Thyroid hormones increase protein synthesis, leading to an increased number of β-receptors, and a reduced myocardial refractory phase.^10^ In a healthy heart, this often manifests as high-output failure.^6^ However, individuals with pre-existing heart conditions are more likely to experience a low cardiac output state due to the hypermetabolic state created during thyroid storm.^4^

In our case, the patient received propranolol as part of the initial regimen used to treat the presentation of suspected thyroid storm. She experienced cardiovascular collapse multiple hours later and subsequent cardiac arrest. The exact etiology of the cardiac arrest is unclear and was most likely multifactorial. Case reports have described cardiac arrest in the setting of thyroid storm, possibly resulting from increased metabolic demand and subsequent heart failure.^2^,^10^ However, the exact mechanism of the precipitating events is often difficult to fully delineate. Several case reports have documented cardiovascular collapse after beta blocker administration, with a possible temporal association particularly with long-acting beta blockers (eg, propranolol). This systematic review only included patients ≥ 18 years of age.^11^

Thionamides and beta blockers have been used successfully within the pediatric population.^12^,^13^ Although beta blockers are often used to help reduce the peripheral effects of increased circulating thyroid hormone, they can be associated with cardiovascular collapse.^6^ Many patients with longstanding hyperthyroidism have some underlying thyrocardiac disease, even if not readily apparent.^8^ In previous cases of beta blocker-induced cardiovascular collapse, nearly all patients had some degree of thyrotoxic cardiomyopathy.^7^ In our case, the patient’s initial chest radiographs showed some mild to moderate cardiomegaly (Image 1). Unfortunately, there are no clear guidelines in this situation since most treatment guidelines for thyroid storm involve the use of beta blockade to reduce the symptoms and clinical manifestations of circulating thyroid hormone.^7^

Beta-blocker toxicity typically presents with bradycardia, hypotension, altered mental status, and hypotension.^14^ In our case, the patient initially received 0.5 mg of IV propranolol approximately seven hours before the cardiac arrest, an additional 1 mg of IV propranolol two hours later, and then 60 mg of PO propranolol another hour later (~ 3.5 hours before the cardiac arrest). Intravenous propranolol has a peak onset of about five minutes, whereas immediate-release oral propranolol has a peak onset of 1–4 hours, according to the US Food and Drug Administration (FDA) product label. The half-life of propranolol is 3–6 hours. In our case, the patient was initially normoglycemic at the outside hospital and was persistently tachycardic, with HR ranging from 130–150 bpm on multiple re-evaluations. Approximately 2–3 hours after the subsequent doses of propranolol were administered, the patient developed worsening hypotension, which had not been noted previously during multiple hours of monitoring.

On arrival at the tertiary-care center, the patient had relative bradycardia at 65 bpm, along with new-onset hypoglycemia, which was noted in association with the patient’s acute change in mental status and subsequent cardiac arrest. Despite initially treating the hypoglycemia with initial improvement, there were multiple recurrences. This ultimately resolved after glucagon administration. The combination of hypoglycemia, bradycardia, hypotension, and altered mental status in our case raised the definite possibility of beta-blocker toxicity as the etiology for the patient’s cardiovascular collapse.

Multiple case reports demonstrate a potential relationship between propranolol administration and cardiovascular collapse.^6^–^8^,^11^ In the setting of potential cardiac instability, clinicians may consider use of short-acting beta blockers as suggested by Abubakar et al (2017), which have the benefit of being easily titratable, particularly in the instance of negative cardiovascular side effects.^8^ Increased metabolic demand and reliance on cardiac output in thyroid storm may increase the susceptibility for cardiovascular collapse secondary to beta blockade. Additionally, combining IV and PO doses of beta blockers or stacking doses at different time intervals may create unintended potentiating effects.

In management of thyroid storm, propranolol has been historically superior to other beta blockers due to its blockade of conversion of T4 to T3. However, esmolol is a short-acting IV beta-1 adrenergic blocker that is easily titratable and may serve as a reasonable alternative to managing thyrotoxicosis.^6^ The half-life of esmolol is nine minutes (2–4 minutes in children).^15^ Landiolol is an ultra-short-acting beta blocker that has also been studied in the management of thyroid storm. It is a beta-1 adrenergic blocker with high cardioselectivity and appears to be cardioprotective, even in situations of pre-existing reduced ejection fraction heart failure.^16^ The half-life of landiolol is 4.5 minutes. This combination may make it a preferable alternative for management of thyroid storm, given the ability for rapid titration. Landiolol has recently received FDA approval for treatment of supraventricular tachycardias, without specific notation for use in thyrotoxicosis. Cardiac arrest has still been reported secondary to landiolol use in thyroid storm treatment, demonstrating the need for judicious use of beta blockers in thyroid storm.^7^

## CONCLUSION

Thyroid storm poses several diagnostic and treatment challenges, with the potential for cardiovascular collapse. Beta blockers are usually indicated but may worsen the hemodynamic status of the patient during the acute phase of thyroid storm and, therefore, should be used with care. Further analysis of the potential use of short-acting and ultra-short-acting beta blockers in thyrotoxicosis is warranted.
